# Transitions in frailty state after kidney transplantation

**DOI:** 10.1007/s00423-020-01936-6

**Published:** 2020-07-20

**Authors:** Evelien E. Quint, Lasse Schopmeyer, Louise B.D. Banning, Cyril Moers, Mostafa El Moumni, Gertrude J. Nieuwenhuijs-Moeke, Stefan P. Berger, Stephan J.L. Bakker, Robert A. Pol

**Affiliations:** 1 Department of Surgery, Division of Vascular and Transplantation Surgery, University Medical Centre Groningen, P.O. Box 30 001, 9700 RB Groningen, The Netherlands; 2 Department of Anaesthesiology, University Medical Centre Groningen, Groningen, The Netherlands; 3 Department of Internal Medicine, Division of Nephrology, University Medical Centre Groningen, Groningen, the Netherlands

**Keywords:** Frailty, Kidney transplantation, Cognition, Mental health

## Abstract

**Purpose:**

Frailty is the body’s failure to return to homeostasis after every day or acute stressful events, causing adverse outcomes. To study its dynamics in kidney transplant recipients (KTR), we determined whether the degree of frailty and its domains are affected by kidney transplantation (KT).

**Methods:**

Between 2015 and 2017, 176 KTR were included. Frailty scores were measured using the Groningen Frailty Indicator (GFI), assessed preoperatively and during follow-up. Transitions in frailty state and changes in the individual domains were determined.

**Results:**

Mean age (±SD) was 51.8 (± 14.1) years, and 63.1% of KTR were male. Thirty patients were considered frail (GFI ≥ 4) at baseline. After a mean follow-up of 22.8 ± 8.3 months, 34 non-frail patients (19.3%) became frail, 125 patients (71.0%) remained the same, and 17 frail patients (9.7%) became non-frail (GFI < 4). In the domain *psychosocial functioning*, 28.4% of the patients had an increase in GFI score after follow-up. Patients who scored a point in the domain *cognition* at baseline had a greater chance of becoming frail (OR 4.38, 95% CI 0.59–32.24).

**Conclusion:**

In conclusion, almost one-fifth of non-frail KTR transitioned to a frail state after their transplantation. These results could be used to predict the impact of KT on frailty course and help with implementing prehabilitation for patients at risk.

**Electronic supplementary material:**

The online version of this article (10.1007/s00423-020-01936-6) contains supplementary material, which is available to authorized users.

## Introduction

Frailty is a frequently occurring physiological condition in today’s aging population. It is the result of aging-associated decline in physical, cognitive, physiological, and immune reserves which leads to a diminished ability to cope with every day or acute stressors [1–3]. Fried et al. [4] have defined frailty as meeting at least three out of five of the following criteria: unintentional loss of weight, low physical activity, exhaustion, low grip strength, and reduced walking speed. An increased inflammatory state, including elevated levels of interleukin 6 and C-reactive protein, has also been reported in frail patients [5]. Next to these physical aspects, limitations in cognitive functioning are an important domain of frailty resulting in unfavorable health outcomes [3].

Preoperatively, approximately one-fifth of kidney transplant recipients (KTR) are considered frail [6]. Frailty in this particular group of patients is associated with an increased risk of immunosuppression intolerance, delayed graft function, and long-term mortality [7–9]. Measuring frailty therefore might aid physicians in the decision to take precautionary measures.

Currently, there are multiple ways to measure frailty [10]. In our center, the Groningen Frailty Indicator (GFI) is used to determine whether a patient is frail or not. The GFI is a multi-domain questionnaire focusing not only on the physical aspect of the patient but also on cognitive and psychosocial domains, which is in contrast to many other frailty indicators [11–14]. It consists of 15 items, involving physical, cognitive, psychological, and social aspects of frailty. A GFI ≥ 4 has previously been demonstrated to be a reliable cut-off value for considering a patient to be frail [15–18]. Unlike other frailty instruments, the GFI proved to be both time and cost efficient, given the short time required to complete the test while covering the most important aspects of frailty [19–21].

Current research is mainly focused on the effect of frailty on kidney transplantation (KT) outcomes [7, 9, 16]. Frailty is dynamic, and intervention such as a KT will most likely have an effect on the degree of frailty [22]. Determining changes in frailty is important to gain insight into the course of frailty after surgery and the individual domains that define these changes. By identifying which domains contribute most to a transition in frailty state, an opportunity to build an individualized frailty prevention program is created. The primary aim of this study was to determine transitions in frailty states after KT over a period of 1 to 3 years. Our secondary aim was to assess which domains and/or patient characteristics contributed most to these transitions.

## Material and methods

### Study design

This study was part of a previously published cohort on frailty in KTR at the University Medical Center Groningen (UMCG), the Netherlands [16]. Two hundred and thirty-three patients were prospectively included during the period of 2015 to 2017. In all patients, the degree of frailty by means of the GFI was measured at admission to the ward prior to kidney transplantation. To determine a transition in frailty state and the individual frailty domains, the questionnaire was obtained a second time by contacting all patients by telephone. In the event that patients were unwilling to discuss this by phone, the questionnaire was sent by conventional mail. A total of 7 patients died during follow-up, and 50 patients were unwilling to participate. One hundred and seventy-six patients (76%) completed the GFI during follow-up and were included in this study. Due to the descriptive character of this study, our institution’s Medical Ethics Committee granted dispensation for the Dutch law regarding patient-based medical research (WMO) obligation (registration no METc2018/050). Patient data were processed and electronically stored according to the declaration of Helsinki - Ethical principles for medical research involving human subjects.

### Frailty score and outcome measures

The GFI consists of 15 questions that cover eight separate domains of functioning: mobility, visual functioning, auditory functioning, nutritional status, comorbidity, cognition, psychosocial functioning, and physical fitness. The GFI questions were answered by the patient with the support of a specially trained nurse. The GFI score was calculated (range 0–15), and patients were considered frail if the outcome resulted in a score ≥ 4 [23] (Supplemental Table S1). The preoperative score was referred to as the index measurement, and the measurement obtained at follow-up was named the follow-up measurement throughout this study.

The primary outcome measure of this study was the transition in frailty state after kidney transplantation. Patients were stratified into three cohorts consisting of 1 year (maximum 12 months), 2 years (minimum 13 to maximum 24), and 3 years (minimum 25 to maximum 36 months) between index and follow-up measurement. For each patient, it was calculated whether there was no transition in frailty state, a transition from non-frail to frail, or a transition from frail to non-frail after kidney transplantation.

Secondary outcome measures were changes within the individual GFI domains. In addition, if a transition had occurred, we determined which variables were most strongly associated with the transition in frailty state.

### Patient data

Collected data included age (years); sex (male/female); type of kidney donor (living/deceased); type of dialysis prior to transplantation (hemodialysis/peritoneal dialysis/hemodialysis and hemodialysis/pre-emptive); duration of dialysis (months); body mass index (BMI; body mass (kg)/height (m)^2^); hypertension (y/n); comorbidities; retransplantation (y/n); length of hospital stay (days); first warm ischemia time (minutes); cold ischemia time (minutes); second warm ischemia time (minutes); delayed graft function (DGF) (y/n) defined as recipients receiving hemodialysis within the first 7 days after transplantation; intensive care admission (y/n); readmission within 30 days of KT (y/n); time between KT and follow-up (months); admission between KT and follow-up (y/n); additional surgical procedures between KT and follow-up (y/n); paired exchange (y/n); and acute rejection (y/n). Comorbidities were assessed using the Charlson Comorbidity Index. The Charlson Comorbidity Index is a weighted score (0–24), which predicts the 1-year mortality of a patient based on the coexisting medical conditions and age [24]. DGF as well as functional DGF was measured in our cohort. Functional DGF is a more suitable way to measure DGF in living donation transplants. We will refer to DGF in the rest of this paper. Acute rejections and diagnostics were in accordance with the Banff Classification of Renal Allograft Pathology reference guide [25].

### Statistical analysis

For the statistical analysis, the Statistical Package for the Social Sciences (IBM SPSS Statistics for Windows, version 23.0. IBM Corp., Armonk, NY, USA) was used. Categorical variables are presented as numbers and percentages. Continuous variables are presented as mean ± standard deviation (SD) for normally distributed variables and as median ± interquartile range (IQR) for skewed variables. Distribution was assessed with the help of a Q-Q plot and a histogram.

Differences between continuous variables were tested with an ANOVA test in case of a normal distribution, and the Mann-Whitney *U* test for a skewed distribution. Differences between categorical variables were assessed using the chi-square test. We analyzed the correlation between transition in frailty state and the before mentioned patient characteristics. We then performed a multivariable analysis using cox regression with non-frail to frail and frail to non-frail as dependent variables. Gender, comorbidities, duration of dialysis, type of kidney donor, and acute rejection were the independent variables in this analysis. These variables were selected based on literature. We also performed a logistic regression with baseline domain measurements as covariates and transition from a non-frail to a frail state as independent. Outcome was adjusted for gender, comorbidities, kidney transplantation type, duration of dialysis, and acute rejection. A *p* value ≤ 0.05 was considered to indicate statistical significance. Estimates of the effects were reported with corresponding 95% confidence intervals.

## Results

### Baseline

Baseline characteristics are presented in Table 1. Mean age of patients was 51.8 ± 14.1 years, of which 63.1% were male. Mean BMI was 26.0 ± 4.3 kg/m^2^. One hundred and four patients (59.1%) were dialysis dependent prior to transplantation and 83% of grafts came from living donors. The mean dialysis duration was 16.7 ± 21.6 months. Twenty-five (14.2%) patients underwent a retransplantation at the time of inclusion. Twelve (6.8%) patients had an acute rejection after kidney transplantation. Mean follow-up time was 22.8 ± 8.3 months. Eighty-two patients (46.6%) were readmitted to the hospital at least once during follow-up of this study.

**Table 1:** Baseline characteristics

Parameter	Number (%), mean ± SD^a^ or median with IQR^b^
Number of patients	176 (100%)
Age (years)	51.8 ± 14.1
Sex	
Male	111 (63.1%)
Female	65 (36.9%)
Type of kidney donor	
Living	146 (83.0%)
Deceased	30 (17.0%)
Type of dialysis	
Hemodialysis	73 (41.5%)
Peritoneal dialysis	29 (16.5%)
Hemodialysis and peritoneal dialysis	2 (1.1%)
Pre-emptive	72 (40.9%)
Duration dialysis (months)	16.7 ± 21.6
BMI^c^ (kg/m^2^)	26.0 ± 4.3
Hypertension	98 (55.7%)
Comorbidities^d^	3.7 ± 1.6
Retransplantation	25 (14.2%)
Paired exchange	14 (8%)
Length of stay (days)	10.1 ± 5.6
First warm ischemia time (minutes)	
Living	3 (3–4)
Deceased	9 (0–16)
Cold ischemia time (minutes)	162 (150–225)
Second warm ischemia time (minutes)	39.0 ± 11.1
Delayed graft function	19 (10.8%)
Acute rejection	12 (6.8%)
Intensive care unit admission	11 (6.3%)
Readmission within 30 days of KT^e^	20 (11.4%)
Time between KT and follow-up (months)	22.8 ± 8.3
Readmission during follow-up	82 (46.6%)
Redo surgery during follow-up	44 (25.0%)

^a^Standard deviation

^b^Interquartile range

^c^Body mass index

^d^According to the Charlson Comorbidity Index, a weighted index which predicts the 1-year mortality by measuring the burden of comorbidities (range from 0 to 19)

^e^Kidney transplantation

### Transition in frailty state

**Fig. 1:**
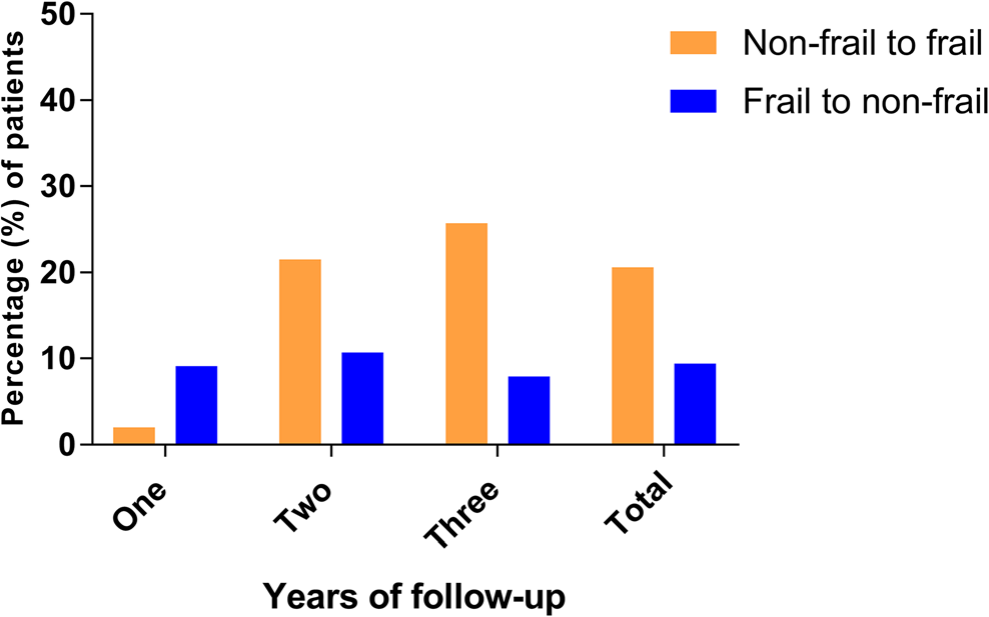
Transition in frailty state during follow-up

In total, 34 non-frail patients (19.3%) became frail, 125 patients (71.0%) maintained their baseline frailty status, and 17 frail patients (9.7%) became non-frail during follow-up. Twelve non-frail patients (24.0%) after 1 year (*N* = 50), 11 non-frail patients (17.7%) after 2 years (*n* = 62), and 11 non-frail patients (19.0%) after 3 years (*n* = 64) became frail. Furthermore, 5 frail patients (10.0%) after 1 year (*N* = 50), 6 frail patients (9.7%) after 2 years (*n* = 62), and 6 frail patients (8.6%) after 3 years (*n* = 64) became non-frail (Table 2; Fig. 1).

**Table 2:** Transition in frailty state

Frail^a^	Number (%)		
Preoperative	30 (17.0%)		
Post-operative	47 (26.7%)		
Years of follow-up	Transition in frailty state		
	From	To	
		Non-frail	Frail
One	Non-frail	29 (58.0%)	12 (24.0%)
	Frail	5 (10.0%)	4 (8.0%)
Two	Non-frail	39 (62.9%)	11 (17.7%)
	Frail	6 (9.7%)	6 (9.7%)
Three	Non-frail	44 (67.2%)	11 (19.0%)
	Frail	6 (8.6%)	3 (5.2%)
Total	Non-frail	112 (63.6%)	34 (19.3%)
	Frail	17 (9.7%)	13 (7.4%)

^a^According to the Groningen Frailty Indicator cut-off of ≥4

### Association between patient characteristics and frailty state

In Table 3, the patient characteristics associated with different transitions in frailty state are shown. Non-frail patients who transitioned to frail were older (55.3 ± 12.7) than frail patients who transitioned to non-frail (51.7 ± 15.6). However, this did not reach statistical significance. There was a significant association between paired exchange and transition in frailty state (*p* = 0.035). After multivariable cox regression analysis, no significant associations were found in the risk of transitioning to another frailty state (Table 4).

**Table 3:** Patient characteristics associated with a transition in frailty state

	Non-frail to frail	No change	Frail to non-frail	*P* value^a^
	*N* = 34	*N* = 125	*N* = 17	
Age (years)	55.3 ± 12.7	50.9 ± 14.3	51.7 ± 15.6	0.227
Sex				
Male	22 (64.7%)	80 (64.0%)	9 (52.9%)	0.659
Female	12 (35.3%)	45 (36.0%)	8 (47.1%)	
Type of kidney donor				
Deceased	7 (20.6%)	22 (17.6%)	1 (5.9%)	0.401
Living	27 (79.4%)	103 (82.4%)	16 (94.1%)	
Type of dialysis				
Pre-emptive	15 (44.1%)	48 (38.4%)	9 (52.9%)	0.629
Hemodialysis	15 (44.1%)	53 (42.4%)	5 (29.4%)	
Peritoneal dialysis	3 (8.8%)	23 (18.4%)	3 (17.6%)	
Hemodialysis and peritoneal dialysis	1 (2.9%)	1 (0.8%)	0 (0.0%)	
Duration of dialysis (months)	21.4 ± 25.9	15.7 ± 20.1	14.81 ± 23.3	0.366
BMI^b^ (kg/m^2^)	25.9 ± 3.2	26.0 ± 4.5	25.9 ± 4.7	0.978
Hypertension	19 (55.9%)	68 (54.4%)	12 (70.6%)	0.450
Comorbidities^c^	3.9 ± 1.7	3.5 ± 1.6	4.1 ± 1.8	0.282
Retransplantation	3 (8.8%)	19 (15.3%)	3 (17.6%)	0.579
Paired exchange	0 (0.0%)	13 (10.4%)	1 (5.9%)	*0.035*
Length of stay	10.2 ± 4.1	10.1 ± 6.2	9.7 ± 5.6	0.180
First warm ischemia time (minutes)	3 (2–4)	3 (2–4)	3 (3–4)	0.710
Cold ischemia time (minutes)	162.5 (153.0–302.0)	162.0 (148.5–202.0)	160.0 (151.5–188.0)	0.964
Second warm ischemia time (minutes)	39.2 ± 10.7	38.7 ± 11.0	40.8 ± 12.9	0.771
Delayed graft function	5 (14.7%)	14 (11.2%)	0 (0.0%)	0.270
Acute rejection	1 (2.9%)	11 (8.8%)	0 (0.0%)	0.127
Intensive care unit admission	2 (5.9%)	6 (4.8%)	3 (17.6%)	0.121
Readmission within 30 days of KT^d^	3 (8.8%)	14 (11.2%)	3 (17.6%)	0.642
Time between KT and follow-up (months)	22.7 ± 8.7	23.0 ± 8.1	22.4 ± 7.9	0.950
Readmission between KT and follow-up	16 (47.1%)	57 (45.6%)	9 (52.9%)	0.849
Surgery between KT and follow-up	8 (23.5%)	32 (25.6%)	4 (23.5%)	0.959

^a^*P* values ≤ 0.05 were considered statistically significant

^b^Body mass index

^c^According to the Charlson Comorbidity Index, a weighted index which predicts the 1-year mortality by measuring the burden of comorbidities (range from 0 to 19)

^d^Kidney transplantation

**Table 4:** Cox regression analysis of factors associated with transition in frailty state

Variable	Non-frail to frail (*n* = 34)	*P* value	Frail to non-frail (*n* = 17)	*P* value^a^
	Hazard ratio (95% CI)		Hazard ratio (95% CI)	
Gender (female)	0.94 (0.07–3.90)	0.53		
Comorbidities (≥ 5 points)^b^	0.88 (0.39–1.97)	0.75	1.86 (0.67–5.12)	0.23
Duration dialysis (≥ 1 year)	1.32 (0.62–2.81)	0.46	1.40 (0.50–3.91)	0.52
Kidney transplantation type	1.21 (0.48–3.04)	0.69	5.64 (0.65–49.20)	0.12
Acute rejection	0.53 (0.72–3.90)	0.53		

^a^*P* values ≤ 0.05 were considered statistically significant

^b^According to the Charlson Comorbidity Index, a weighted index that predicts 1-year mortality by measuring the burden of comorbidities (range 0–19)

### Change per domain

**Fig. 2:**
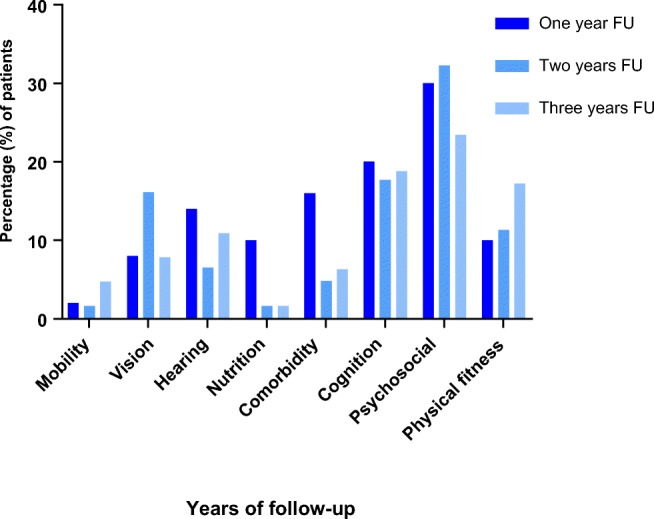
Patients with an increase in GFI score during FU, per domain. GFI, Groningen Frailty Indicator; FU, follow-up

The changes in GFI score per domain are shown in Fig. 2 and Supplemental Table S2. In the domains *cognition*, *psychosocial*, and *physical fitness*, 18.8%, 28.4%, and 13.1%, respectively, of the patients showed an increase in GFI score. In the domain *nutrition*, 10% of the patients showed an increase in GFI score in the 1-year follow-up group. In the 2-year follow-up group and the 3-year follow-up group, 1.6% of patients showed an increase in GFI. In the domains *mobility* and *nutrition*, 94.9% and 90.3%, respectively, of patients had no change in GFI score.

### Association between baseline domain score and the transition from a non-frail to a frail state

In Table 5, the associations between baseline domain score and becoming frail are shown. Patients who scored a point in the domain *cognition* at baseline had a greater chance of becoming frail (OR 4.38, 95% CI 0.59–32.24). Patients who scored a point in the domain *comorbidity* at baseline had a greater chance of becoming frail (OR 1.61, 95% CI 0.35–7.51).

**Table 5:** The effect of the individual frailty domains at baseline on the transition from a non-frail to a frail state

Frailty domain	Non-frail to frail (*n* = 34) Odds ratio (95% CI)
Mobility *Mobility*^*Ω*^	0.00 (0.00–0.00) 0.00 (0.00–0.00)
Vision *Vision*^*Ω*^	0.33 (0.04–2.62) 0.34 (0.04–2.82)
Hearing *Hearing*^*Ω*^	0.51 (0.06–4.20) 0.54 (0.06–4.70)
Nutrition *Nutrition*^*Ω*^	0.92 (0.19–4.48) 1.10 (0.21–5.34)
Comorbidity *Comorbidity*^*Ω*^	1.61 (0.35–7.51) 1.50 (0.30–7.02)
Cognition *Cognition*^*Ω*^	4.38 (0.59–32.24) 3.44 (0.43–27.34)
Psychosocial *Psychosocial*^*Ω*^	0.79 (0.32–1.98) 0.74 (0.29–1.88)
Physical fitness *Physical fitness*^*Ω*^	0.67 (0.31–1.43) 0.72 (0.33–1.60)

^a^*P* values ≤0.05 were considered statistically significant

^Ω^Outcomes adjusted for gender, comorbidities, kidney transplantation type, duration of dialysis, and acute rejection

## Discussion

This study shows the dynamic course of frailty after KT during the first 3 years of follow-up, wherein almost one-fifth of non-frail patients became frail. The frailty domains *cognition* and *psychosocial functioning* seem to be the main contributors to this transition. Though there were transitions in frailty state, there was no significant association with outcomes such as delayed graft function or the number of hospital readmissions.

Our results highlight the importance and advantage of including a broad range of domains when measuring frailty. Frailty is multifactorial, and it is necessary to be aware of all the factors that can lead to an increase in frailty to focus on mitigation efforts. Even if a patient is not considered frail, the individual components of frailty could lead to adverse outcomes [10, 26]. The GFI provides a holistic perspective of frailty, including both physical and psychological components as subgroups, which are often missing in other frailty tools, making it more versatile [23].

In our study, a positive baseline *cognition* score was associated with a 4 times greater chance of becoming frail. This shows the importance of customized intervention, such as prehabilitation, to avoid further decline. The domain *psychosocial functioning* also contributed to a transition from a non-frail to frail state. However, this was not shown to be significant in the logistic regression. At baseline, patients are not struggling psychosocially, but rather that they face problems after kidney transplantation.

Until now, studies have mainly looked at the relationship between depressive symptoms, frailty, and kidney transplantation. It was found that having depressive symptoms led to an increased risk of becoming frail after KT [26, 27]. Likewise, cognitive function has only been studied as a component separate from frailty in the kidney transplant population. Recent research showed that frail patients, according to Fried physical frailty phenotype, have an increased cognitive performance (Modified Mini-Mental State Investigation) in the short term following KT but show declines in cognition in the long term [28]. Based on these data, psychosocial and cognitive factors are apparently of little known influence but have a significant impact on frailty and hence deserve extra attention. It is therefore striking that among transplantation health care professionals only 2.2% believe that psychosocial status is of importance when defining frailty [29]. Additionally, in our center, no program exists to support the psychosocial and cognitive wellbeing of KT candidates. A proactive and preventative measurement should be implemented to combat the negative impact of this domain and increase awareness.

A pilot focusing on prehabilitation among KTR suggested that significant improvement in physical exercise occurs when a preventative program, consisting of physical therapy and at-home exercises, is implemented [30]. Furthermore, cognitive training and exercise training have been shown to possibly prevent cognitive decline in hemodialysis patients [31]. Ultimately, creating a patient-tailored post-surgical program including psychosocial aspects may decrease frailty. This in turn may improve the overall health and wellbeing of the patient and reduce the burden on the healthcare system in the long run. This could have positive financial and capacity effects on a healthcare system that is already under pressure. Prevention and battling against decline in the cognitive and mental health of KTR should become a standard part of transplant care.

Prior studies have shown that there are numerous factors which contribute to becoming frail. In this study, we found that patients who transitioned from a non-frail state to a frail state were older than patients who transitioned from a frail state to a non-frail state. When people are older at baseline, the likelihood of being/becoming frail increases with the corresponding risk of becoming more frail while aging in general [1, 22, 32]. All patients have end-stage renal disease (ESRD) and the majority requires dialysis, which in itself is associated with an increased risk of being frail [33]. More than one-third of patients with ESRD is frail [34]. Patients with advanced chronic kidney disease often have a low energy intake and are less active than healthy controls. Additionally, there is an increased inflammatory state caused by elevated levels of pro-inflammatory cytokines in chronic kidney disease [35]. The frailty trajectory among this group of patients is, however, extremely variable with almost the same number of patients improving as deteriorating in frailty score [36]. In the case of kidney transplantation, patients undergo a major surgical procedure associated with a disruption of the vital homeostasis from which the body has to recover. Furthermore, the body has to adapt to the new immunosuppressive drug regimen. These factors increase the risk for a kidney transplant recipient to become more frail over time, compared with community-dwelling older adults who do not endure these changes.

Almost half of the patients which transitioned from a non-frail to a frail state were pre-emptively transplanted. One would expect pre-emptive patients to be less at risk of becoming frail, given that dialysis can have a great impact on frailty [33]. Prior to transplantation, many pre-emptive patients do not feel ill, though they are living with end-stage kidney disease. After receiving a transplant, they are faced with new problems like infections, complications, stress, and sometimes guilt, all adding to the risk of becoming frail or creating a sense of vulnerability which negatively affects psychosocial and cognitive wellbeing. In contrast, dialysis patients often already experience these issues before the transplantation. Post-transplantation, they do not experience major changes or improve in psychosocial state now that they have a functioning kidney [37]. These changes and effects are currently underexposed in the majority of the kidney transplant programs, including ours. More attention is necessary, including the implementation of a possible intervention.

Interestingly, a part of our population of KTR seemed to improve in frailty (frail to non-frail state) during follow-up. Despite the enormous impact that a KT has on the physical and cognitive reserves of a recipient, the health benefit compared with dialysis or end-stage renal disease is extensive. Herein lies the crux, since a proper patient selection can play an important role in the expected outcome. Which patient will have maximum benefit from a transplant and which will subsequently be hindered by an increase in frailty causing him or her to no longer function at the pre-transplant level? These issues are difficult to answer but must continue to receive attention in view of the increasing aging of the population.

This study has some limitations that need to be addressed. First, contacting the patient by phone creates a risk of bias. There is a risk that patients do not give all the correct information because an immediate response is required and face to face contact is missing. Also, patients may feel ashamed to report psychological and physical problems or restrictions. To minimize this risk, patients were given sufficient time to think about their answers and if they were not willing to give a response by phone, they received the questionnaire by mail and were offered a follow-up telephone consultation. Our response rate, using this approach, was 75.5% which is in accordance with other survey studies and resulted in a reliable selection to perform our analyses [38–41]. Second, the GFI is a self-reported questionnaire. Because of differing personalities, pain thresholds, and several coping strategies, a variation in reporting can occur, leading to an under- or overestimation. However, we focused on a transition in frailty within the individual and not on a static frailty measurement. Third, the GFI has not been used as an evaluative measurement instrument until now, only as a screening instrument. The GFI is a widely used, validated screening instrument among various patient categories and can, by its compact and simple design, be filled in by the patient in a relatively short amount of time while covering many aspects of frailty. Fourth, 25 patients had undergone a retransplantation during follow-up which could have clouded the effect of KT on frailty transition. However, the degree of transition in this group was comparable to the entire cohort. The domain *psychosocial functioning* contributed most to this increase in the retransplantation group. Fifth, measuring frailty at fixed time points and measurements during acute events would have provided a more detailed analysis of transitions in frailty. However, we believe that this study still accurately represents transitions in frailty state that occur during follow-up.

## Conclusion

In conclusion, almost one-fifth of the non-frail kidney transplant patients transitioned to a frail state during the follow-up period of 1 to 3 years after transplantation. Cognitive functioning and psychosocial wellbeing were particularly affected and thereby contributed the most to a transition in becoming frail. Prevention and battling against the decline of the cognitive and mental health of KTR should become a standard part of transplant care. By preoperatively identifying patients at risk of becoming more frail after transplantation, health care providers might be given a tool to decide which specific prehabilitation program and post-surgical therapy could be most beneficial in improving long-term patient and graft outcome.

## Electronic supplementary material

ESM 1 (DOCX 19 kb)

## Data Availability

The datasets generated during and/or analyzed during the current study are available from the corresponding author on reasonable request.
