# Supplementary material for: Transitions in frailty state after kidney transplantation
Source: Langenbecks Arch Surg. 2020 Jul 20;405(6):843–50. doi: 10.1007/s00423-020-01936-6 (PMC7471154; doi:10.1007/s00423-020-01936-6)
Supplement: Supplementary file 1 — (DOCX 19 kb) [file 423_2020_1936_MOESM1_ESM.docx]

**Supplemental Tables**

**Supplemental Table 1.** The Groningen Frailty Indicator (GFI)

|  | YES | NO |  |
| --- | --- | --- | --- |
| **Mobility**  Can the patient perform this task without any help? (using tools like walking sticks, wheelchairs or walker is regarded as independent) |  |  |  |
| 1. Go shopping | **0** | **1** |  |
| 2. Walk around outside (around the house or to neighbours) | **0** | **1** |  |
| 3. Dressing and undressing | **0** | **1** |  |
| 4. Toilet visit | **0** | **1** |  |
| **Vision**  5. Does the patient experience problems in daily life by poor vision? | **1** | **0** |  |
| **Hearing**  6. Does the patient experience problems in daily life by poor hearing? | **1** | **0** |  |
| **Nutrition**  7. Has the patient involuntarily lost weight (≥6kg) in the past 6 months (or ≥3 kg in one month) | **1** | **0** |  |
| **Comorbidity**  8. Does the patient currently use four or more different types of medication? | **1** | **0** |  |
|  | **Yes** | **No** | **Sometimes** |
| **Cognition**  9. Does the patient currently has complaints about his/her memory (or has a history of dementia) | **1** | **0** | **0** |
| **Psychosocial**  10. Does the patient sometimes experience emptiness around him/her? | **1** | **0** | **1** |
| 11. Does the patient sometimes miss people around him? | **1** | **0** | **1** |
| 12. Does the patient sometimes feel abandoned? | **1** | **0** | **1** |
| 13. Has the patient recently felt sad or depressed? | **1** | **0** | **1** |
| 14. Has the patient recently felt nervous or anxious? | **1** | **0** | **1** |
|  |  |  |  |
| **Physical fitness**  15. Which grade would the patient give its physical fitness (0-10, ranging from very bad to good ) 0-6=1 7-10= 0 | **1** | **0** |  |
| **TOTAL SCORE GFI** |  | | |

A score of four or more indicates a risk for frailty.

**Supplemental Table 2.** Change in GFI score over time, per domain

| GFI domain and  years of follow-up | Increase in GFI score | No change in GFI score | Decrease in GFI score |
| --- | --- | --- | --- |
| Mobility  *One year* (N=50)  *Two years (N=62)*  *Three years (N=64)*  Total (N=176) | 1 (2.0%)  1 (1.6%)  3 (4.7%)  5 (2.8%) | 47 (94.0%)  60 (96.8%)  60 (93.8%)  167 (94.9%) | 2 (4.0%)  1 (1.6%)  1 (1.5%)  4 (2.3%) |
| Vision  *One year* (N=50)  *Two years (N=62)*  *Three years (N=64)*  Total (N=176) | 4 (8.0%)  10 (16.1%)  5 (7.8%)  19 (10.8%) | 42 (84.0%)  50 (80.7%)  56 (87.5%)  148 (84.1%) | 4 ( 8.0%)  2 (3.2 %)  3 (4.7%)  9 (5.1%) |
| Hearing  *One year* (N=50)  *Two years (N=62)*  *Three years (N=64)*  Total (N=176) | 7 (14.0%)  4 (6.5%)  7 (10.9%)  18 (10.2%) | 42 (84%)  57 (91.9%)  54 (84.4%)  153 (86.9%) | 1 (2.0%)  1 (1.6%)  3 (4.7%)  5 (2.9%) |
| Nutrition  *One year* (N=50)  *Two years (N=62)*  *Three years (N=64)*  Total (N=176) | 5 (10.0%)  1 (1.6%)  1 (1.6%)  7 (4.0%) | 42 (84.0%)  56 (90.3%)  61 (95.3%)  159 (90.3%) | 3 (6.0%)  5 (8.1%)  2 (3.1%)  10 (5.7%) |
| Comorbidity  *One year* (N=50)  *Two years (N=62)*  *Three years (N=64)*  Total (N=176) | 8 (16.0%)  3 (4.8%)  4 (6.3%)  15 (8.5%) | 40 (80.0%)  56 (90.3%)  57 (89.1%)  153 (86.9%) | 2 (4.0%)  3 (4.8%)  3 (4.7%)  8 (4.6%) |
| Cognition  *One year* (N=50)  *Two years (N=62)*  *Three years (N=64)*  Total (N=176) | 10 (20.0%)  11 (17.7%)  12 (18.8%)  33 (18.8%) | 39 (78.0%)  49 (79.0%)  52 (81.3%)  140 (79.5%) | 1(2.0%)  2 (3.2%)  0 (0.0%)  3 (1.7%) |
| Psychosocial  *One year* (N=50)  *Two years (N=62)*  *Three years (N=64)*  Total (N=176) | 15 (30.0%)  20 (32.3%)  15 (23.4%)  50 (28.4%) | 28 (56.0%)  33 (53.2%)  43 (67.2%)  104 (59.1%) | 7 (14.0%)  9 (14.5%)  6 (9.4%)  22 (12.5%) |
| Physical fitness  *One year* (N=50)  *Two years (N=62)*  *Three years (N=64)*  Total (N=176) | 5 (10.0%)  7 (11.3%)  11(17.2%)  23 (13.1%) | 33 (66.0%)  38 (61.3%)  35 (54.7%)  106 (60.2%) | 12 (24.0%)  17 (27.4%)  18 (28.1%)  47 (26.7%) |

^a^ Groningen Frailty Indicator
